# Norms for a German version of the Phenomenological Control Scale*:* measuring the capacity for creating and controlling experience

**DOI:** 10.1093/nc/niag073

**Published:** 2026-10-31

**Authors:** Moritz Schiltenwolf, Marie Luise Schreiter, Peter Lush

**Affiliations:** LMU Munich, Department of Psychology, Leopoldstraße 13, 80802 Munich, Germany; Max Planck Institute for Biological Cybernetics, Max-Planck-Ring 8 - 10, 72076 Tübingen, Germany; University of Sussex, Falmer Hill, Brighton BN1 9RH, United Kingdom

**Keywords:** phenomenological control, imaginative suggestion, individual differences

## Abstract

The ability to create experiences which misrepresent reality and thereby meet strategic goals is a stable trait that varies substantially in the general population. The capacity for such phenomenological control can be measured by response to imaginative suggestions for a variety of experiences, e.g. hallucination in various modalities, amnesia, paralysis, or motor actions, which are experienced as involuntary. Here, we present norms for a German translation of the Phenomenological Control Scale [Lush *et al.* (The Phenomenological Control Scale: measuring the capacity for creating illusory nonvolition, hallucination, and delusion. *Collabra: Psychology* 2021;7:29542)]. Data were collected from a representative sample of native-German speakers (*N* = 299) using a single-session online screening procedure. We show that the translated scale is psychometrically sound, and in line with the results of the original scale in terms of mean scores, distribution, and internal consistency. All analyses, code, data, materials, and the full questionnaire script are made freely available. Other researchers in the field of psychology are welcome to access and encouraged to use the German norms for the Phenomenological Control Scale, e.g. to assess the degree to which trait phenomenological control may be involved in other psychological effects.

## Introduction

The capacity to alter subjective experience in line with one’s beliefs and goals (phenomenological control; alternatively, “the ability to alter experience”; Dell 2021, 2024) can be measured by response to imaginative suggestions across multiple sensory and motor modalities. Although phenomenological control has historically been investigated primarily within the context of hypnosis research (Sheldrake and Dienes 2026), the hypnotic context (e.g. an induction procedure which aims to produce a special “state”) is not required for response to imaginative suggestion (Hull 1933; Braffman and Kirsch 1999). Because the use of phenomenological control is not limited to the particular social context of hypnosis, it may be used to fulfill particular needs or perceived social roles in a variety of situations. For example, to produce experiences in response to experimental demand characteristics (Michael et al. 2012; Dienes et al. 2020; Corneille and Lush 2023; Lush et al. 2026) or to mentally distance oneself from physical or emotional pain (Dell 2021). There is therefore a need for tools which can support research into individual differences in this trait capacity, e.g. to measure the degree to which psychological effects are differentially reported by those high or low in phenomenological control (e.g. Lush et al. 2020, 2026).

The Phenomenological Control Scale (PCS), originally developed in English by Lush et al. (2021), provides a standardized method for assessing this capacity. The PCS is an adaptation of the Sussex-Waterloo Scale of Hypnotisability, itself a modification of the Waterloo-Stanford Group Scale of Hypnotic Suggestibility, Form C (Bowers 1993), and therefore ultimately derived from the Stanford Hypnotic Susceptibility Scale, Form C (Weitzenhoffer and Hilgard 1962). The PCS differs from the Sussex-Waterloo Scale of Hypnotisability in that all references to a hypnotic context have been removed (e.g. references to sleep and relaxation or a special state), and the scale is presented as a test of a capacity to generate particular experiences, rather than as a hypnotic induction procedure. The scale consists of 10 items involving suggestions across a range of domains, including amnesia, auditory, visual, gustatory, tactile, motor, and paralysis experiences. Participants rate their subjective responses on a scale from 0 to 5, yielding an average PCS score that reflects the extent to which individuals can generate the suggested experiences. Score distributions are approximately Gaussian and broadly comparable to those obtained from classic hypnotic suggestibility scales. While non-hypnotic imaginative suggestions historically have been shown to produce a slightly weaker response than “hypnotic” procedures (Martin and Dienes 2019), PCS norms show higher scores than those for the hypnosis scale from which it is adapted driven by a shift of low scorers toward the mid-range. This shift may be attributable to the reframing of imaginative suggestion as a capacity or skill, which may reduce the impact of negative preconceptions associated with hypnosis (e.g. the myth that responding to suggestion implies that one is “suggestible,” or unusually socially compliant; Dienes et al. 2020; Lynn et al. 2020). The PCS inherits practical advantages offered by the Sussex-Waterloo Scale of Hypnotisability over the scales on which it is based, notably, substantially shorter administration time and modifications which allow online delivery (Palfi et al. 2020; Imaizumi and Suzuki 2025). The PCS can be administered online in around 30 min using common online questionnaire platforms (e.g. SocSci, RedCap, etc). Norms for a Japanese adaptation of the PCS (PCS-J; Imaizumi and Suzuki 2025) show similar properties to the original scale norms.

The present study aimed to develop and validate a German version of the PCS (PCS-GER) to facilitate future research on the effects of phenomenological control in native German-samples. Further, we aimed to verify the feasibility of online administration, following prior work demonstrating the suitability of recorded audio instructions for remote delivery (Palfi et al. 2020; Imaizumi and Suzuki 2025). To this end, the PCS script was presented via audio recordings in German, and the data was collected online to evaluate the psychometric properties of the PCS in a German-speaking sample.

## Methods

### Participants

A total of *N* = 357 participants were recruited online for the study. The preregistered sample size was 300 participants set as a minimum recruitment target to account for potential exclusions and to obtain robust information about the usability and psychometric performance of the scale (Boateng et al. 2018). In the end, 262 of the participants were recruited using the participant pool provider Prolific; the remaining 95 participants were recruited via the local study platform SONA of the University of Tübingen.

To assess response quality, we excluded participants who completed the survey in less time than was considered realistically necessary to listen to the instructions (*n* = 57). At the end of the survey, participants also indicated whether they had completed the items meaningfully and conscientiously. Two participants reported that they had not responded meaningfully, one of whom had already been excluded based on completion time. In total, 58 participants were excluded. These criteria were applied in addition to the preregistered exclusion criterion (https://osf.io/b7yfx), which excluded participants who did not complete all survey items.

The 299 participants (185 female, 112 male, and 2 diverse) included in the analysis ranged in age from 18 to 72 years (*M* = 31, SD = 12). Participants were compensated either with course credit or £3.75 for their participation. Participants provided informed consent prior to participation. The collected data were anonymous.

### Materials and Translation

Participants completed this online study on their own computers; they were asked to do it in a quiet room with no distractions and to wear headphones for the whole duration of the study.

The stimulus material consisted of the PCS, translated from English into German using official guidelines for questionnaire translation (Duc et al. 2025). The initial translation from the original script (English) into German was translated and checked by three independent bilingual translators (Walde and Völlm 2023). Two of the translators were aware of the concepts the questionnaire intends to measure to best resemble the original instrument. Another naïve German speaker without a background in psychology or neuroscience was asked to read the translation and make small changes if necessary to reflect the nuances of the spoken German language. Finally, the text was back-translated by the researchers to ensure the accuracy of the translation and checked by a native English speaker (also part of the research team) to avoid misunderstanding and unclear wording. The translated materials can be found in the Supplemental Materials B and C.

The script was then audio recorded by a male native German speaker. Within the study, the first part of the audio was presented via a YouTube video with a black screen and only the audio playing except for an image of three balls (red, green, and blue) being shown at the end (see “negative visual hallucination”). The rest of the text was presented via audio snippets. All audio files, images and the full text can be found on GitHub (Schiltenwolf et al. 2026; https://github.com/Schiwo/The-German-Phenomenological-Control-Scale). The data was collected online via SoSciSurvey (www.soscisurvey.de).

The translated scale consisted of the same 10 items as the original PCS in English. There are four ideomotor suggestions (i.e. suggestions for experience of involuntary movement): hand lowering (a suggested experience of a heavy object in the participant’s outstretched hand) and moving hands together (a suggested experience of a “magnetic” force pulling the participant’s outstretched hands together). There are two ideomotor items involving suggestions for an inability to move: arm rigidity (the arm is so rigid that it cannot be bent) and arm immobilization (the arm cannot be lifted from the participant’s lap). There is one post-session suggestion in which participants are told that they will press the space bar six times in a row but will not remember being told to do so. The remaining items are cognitive–perceptual suggestions: experience of a mosquito (tactile or auditory experience), music (hearing Happy Birthday played), negative visual hallucination (participants are shown a slide of three colored balls and told they can see only two), amnesia (participants are unable to remember anything from the session until that point), and taste (suggestions for experiences of sweet and sour tastes).

The “subjective” scale records experience (e.g. how much it felt like there was a magnetic force drawing the hands together) on a Likert scale (from 0–5). Responses were recorded by participants using their computer mouse, on a presented Likert rating scale.

**Figure 1:**
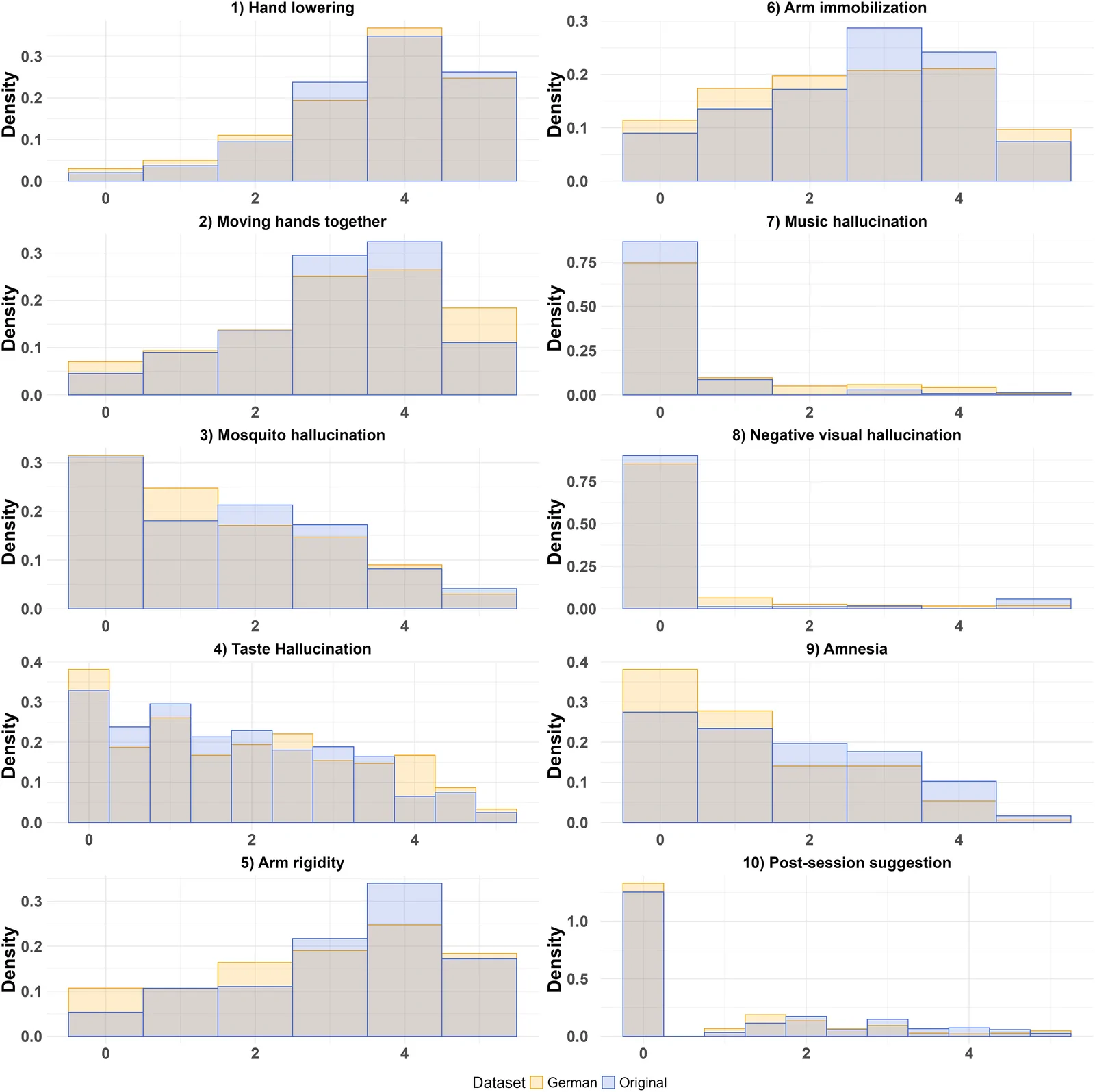
Item-level distribution comparisons. The distribution of PCS scores in the German dataset (orange) is compared with that of the original dataset (blue), shown using histograms (bars). Item 4 and 10 are presented in 0.5 increments since they were calculated as the arithmetic (item 4) or geometric (item 10) mean of two reports.

### Procedure

Participants were recruited via Prolific or registered through the university SONA system. Upon accessing the study link, participants were asked to provide demographic information and were given the opportunity to provide informed consent before proceeding to the experiment.

A sound check was conducted at the beginning of the session to ensure that the audio volume was adequate and comfortable. Participants were then instructed to start the video when they felt ready. The video began with a brief imagination task designed to familiarize participants with the procedure, followed by the presentation of the imaginative suggestions.

After the audio stimuli had concluded, participants were asked to rate the strength of each of the 10 individual experiences using a 6-point Likert scale ranging from 0 to 5, with scale anchors translated from the PCS. The response scale was explained prior to each exercise. The median study duration was 16 min (minimum 15.5 min; maximum 65.4 min). At the end of the session, all participants were debriefed and informed about the purpose and nature of the study.

### Analyses

At the item level, the following analyses were conducted. We investigated the item distribution indicators compared with the originally published data set by Lush et al. (2021), excluding the retest data. Indicators of internal consistency were calculated, including Cronbach’s Alpha and Cronbach’s Alpha if item deleted. 95% confidence intervals were estimated using a bootstrapping procedure with 5000 iterations. Finally, item-to-PCS score correlations were computed.

At the scale level, PCS-GER scores for each participant were calculated, as specified in the preregistration document, as the mean across all 10 scale items. Consistent with the original scale, two items were derived from pairs of subjective responses to separate suggestions. Item 4 (taste hallucination) was computed as the arithmetic mean of responses to suggestions involving experiences of sweet and sour taste, whereas Item 10 (post-hypnotic suggestion) was computed as the geometric mean of the reported urge to press the space bar and the reported amnesia following the suggestion to forget the previous instruction. In addition, the same distribution metrics as those used for the individual items (mean, standard deviation, skewness, and kurtosis) were calculated for the scale scores and compared with the original data (Lush et al. 2021; retrieved from https://osf.io/4x25a).

**Table 1:** Item score distribution indicators for the German validation sample (in this order: mean, standard deviation, item-drop Cronbach’s alpha with 95% CI, and corrected item-to-total correlation).

**Item**	***M***	***SD***	**α** _**drop**_ **[95% CI]**	***R*** _**corrected**_ **[95% CI]**
1. Hand lowering	3.6	1.3	0.68 [0.62, 0.73]	0.53 [0.44, 0.62]
2. Moving hands together	3.1	1.5	0.70 [0.64, 0.75]	0.42 [0.31, 0.51]
3. Mosquito hallucination	1.5	1.4	0.70 [0.64, 0.77]	0.41 [0.32, 0.50]
4. Taste hallucination	1.9	1.5	0.71 [0.65, 0.75]	0.37 [0.28, 0.46]
5. Arm rigidity	2.9	1.6	0.66 [0.59, 0.72]	0.60 [0.53, 0.67]
6. Arm immobilization	2.5	1.5	0.67 [0.61, 0.73]	0.55 [0.47, 0.63]
7. Music hallucination	0.6	1.2	0.72 [0.67, 0.77]	0.23 [0.11, 0.35]
8. Negative visual hallucination	0.3	1.0	0.73 [0.67, 0.77]	0.19 [0.08, 0.29]
9. Amnesia	1.2	1.3	0.71 [0.65, 0.75]	0.35 [0.24, 0.45]
10. Post-hypnotic suggestion	0.8	1.3	0.74 [0.69, 0.78]	0.14 [0.04, 0.25]

CIs were calculated using bootstrapping procedures. Corrected item-to-total correlation means correlating an item with the scale score excluding that specific item’s contribution.

**Figure 2:**
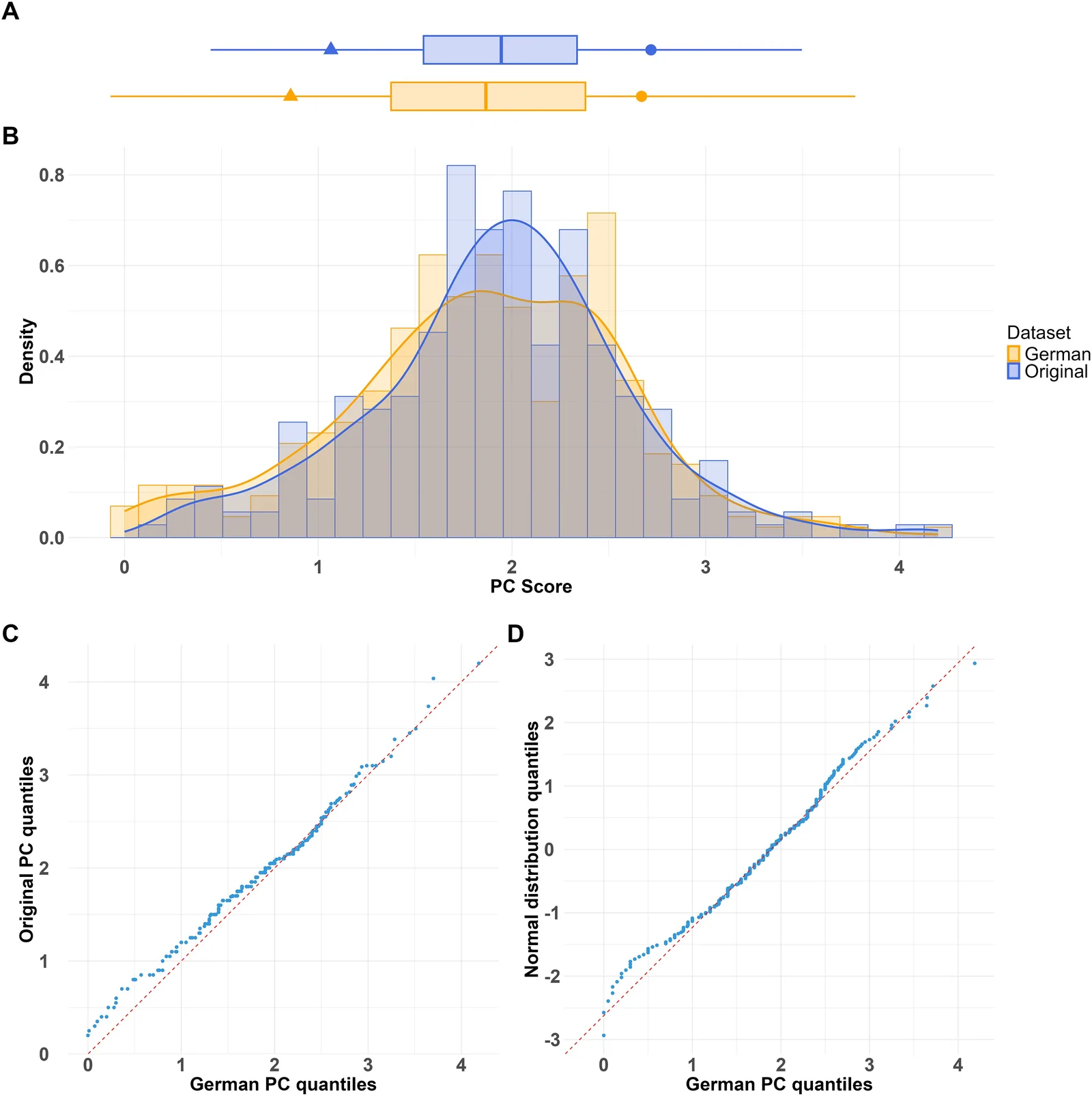
PCS score distribution comparisons. In the top half of the figure, the distribution of PCS scores in the German dataset (orange) is compared with that of the original dataset (blue). Panel (A) shows boxplots with the box displaying the interquartile range (IQR), the bold line displaying the median, whiskers displaying 1.5 IQR, and the 10th and 90th quantiles displayed by triangles and points and panel (B) shows histograms with smoothed density functions. In the bottom half, two quantile–quantile (Q–Q) plots display the quantiles of the PCS score in the German sample data on the *x*-axis against the quantiles of the PCS score in the original dataset (bottom left; panel C) and against the quantiles of a theoretical normal distribution (bottom right; panel D).

## Results

The mean scale PCS-GER score was 1.8 (SD = 0.7), closely matching the mean score reported for the original PCS (*M* = 1.9, SD = 0.7). Across all ten items, Cronbach’s Alpha showed adequate internal consistency, α = 0.73, 95% CI [0.67, 0.77], comparable to the internal consistency for the original scale (α = 0.68, 95% CI [0.60, 0.74]). The PCS-GER score revealed a mean correlation with the ten individual items of *R* = 0.53 (original data: *R* = 0.51). A visual inspection of the density histograms at the item level for the two samples (see Fig. 1) shows similar distributions for each item. Distribution parameters are shown in Table 1. A more detailed descriptive comparison between scale- and item-level statistics of the validation samples of the PCS-GER and the original PCS (Lush et al. 2021) can be found in the Supplemental Material A.

Figure 2 displays the PCS score distribution of the German version against the distribution of the original data and a theoretical normal distribution, showing a similar approximately normal distribution for each scale. Additionally, a two-sample Kolmogorov–Smirnov test was conducted to compare the distribution of PCS scores for the Original and German sample. The test revealed no statistically significant difference between the distributions, *D* = 0.090, *P* = .232, indicating that the largest absolute difference between the empirical cumulative distribution functions of the two samples was 9%. A comparison of the latent factor structure between the German and the original data can be found in the Supplemental Material B.

## Conclusion

Here, we present the German version of the PCS (PCS-GER), designed to measure the trait ability to generate perceptual experiences in response to external suggestions. This version represents a direct translation of the original English PCS developed by Lush et al. (2021) conducted in accordance with established guidelines for questionnaire translation (Duc et al. 2025). To validate that the German version of the PCS captures the same construct as the original scale, a large sample was collected.

Distribution parameters were compared with those reported in the validation study of the original PCS (Lush et al. 2021). The distribution of PCS scores obtained with the PCS-GER showed similar properties to the distribution observed in the original English-language PCS sample. Internal consistency was also comparable to that reported in the original study. Inspection of individual item distributions indicated that they reflected the expected variation in item difficulty and item score distributions. Finally, these distributions closely matched those observed in the validation sample of the original PCS.

The present findings are consistent with previous studies that administered measures of responsiveness to imaginative suggestions online. In particular, the distribution of PC scores closely matched that reported for the Japanese translation of the PCS (Imaizumi and Suzuki 2025) with comparable distribution parameters, indicating that comparable PC score distributions can be obtained across different language versions of the PCS. Notably, whereas the original PCS validation sample was assessed in the laboratory, both the present study and the Japanese validation study collected data online. This pattern is consistent with the observations of Palfi et al. (2020), who likewise reported that online administration of measures assessing responsiveness to imaginative suggestions yields outcomes comparable to those obtained with traditional in-person administration.

A limitation of the present study is that neither test–retest reliability nor construct validity was assessed, e.g. through within-subject comparisons with related constructs, such as hypnotizability (Lush et al. 2018; Palfi et al. 2020). Thus, although the current findings provide initial evidence for the instrument’s psychometric performance, they do not constitute a comprehensive psychometric validation of the PCS-GER. However, both the original PCS and the Japanese version demonstrated good test–retest reliability and convergent validity (Lush et al. 2021; Imaizumi and Suzuki 2025). Because the PCS-GER retained the original items, response format, and underlying construct, there was no strong theoretical reason to expect substantial differences in temporal stability and construct validity. Nevertheless, translation and cultural adaptation may influence how individual items are interpreted. Future studies should therefore address this limitation by assessing test–retest reliability as well as convergent and discriminant validity in a German sample evaluated for phenomenological control ability.

In summary, the present findings demonstrate that the German translation, PCS-GER, exhibits psychometric properties highly comparable to those of the original English-language PCS. Accordingly, the PCS-GER represents an appropriate instrument for assessing trait phenomenological control in German-speaking populations.

## Supplementary Material

Supplementary_materials_niag073

## Data Availability

The data, materials, and analysis scripts are available at https://github.com/Schiwo/The-German-Phenomenological-Control-Scale. The preregistration for the sample size rational, outlier handling, and the main analysis is available at https://osf.io/b7yfx.
