# Supplementary material for: Norms for a German version of the Phenomenological Control Scale: measuring the capacity for creating and controlling experience
Source: Neurosci Conscious. 2026 Oct 31;2026(1):niag073. doi: 10.1093/nc/niag073 (PMC13723268; doi:10.1093/nc/niag073)
Supplement: Supplementary file 1 [file supplementary_materials_niag073.zip › NoC_Supplementary Material_Norms for a German version of the Phenomenological Control Scale_rev_clear.docx]

**Supplementary Materials for:**

**Norms for a German version of the Phenomenological Control Scale*:* Measuring the capacity for creating and controlling experience**

Moritz Schiltenwolf*^1 [^[^0000-0002-6539-5512^](https://orcid.org/0000-0002-6539-5512)^]0000-0002-6539-5512^

Marie Luise Schreiter*^2[^[^0000-0002-4596-9195^](https://orcid.org/0000-0002-4596-9195)^],^

Peter Lush^3^ ^[^[^0000-0002-0402-1699^](https://orcid.org/0000-0002-0402-1699)^],^

1 LMU Munich, Germany

2 Max Planck Institute for Biological Cybernetics, Tübingen, Germany

3 University of Sussex, Brighton, United Kingdom

*Contributed equally

Correspondence should be addressed to

Marie Luise Schreiter

Max-Planck-Institut für biologische Kybernetik

Max-Planck-Ring 10

72076 Tübingen, Germany

[m.luise.schreiter@tuebingen.mpg.de](mailto:m.luise.schreiter@tuebingen.mpg.de)

**Supplementary Material**

**A) Comparison of distribution parameters between the samples**

In the German validation sample, the distribution of the PC score was approximately symmetric (γ_1_ = -0.20; original data skew γ_1_ = 0.01) and a marginally heavier-tailed distribution than the normal distribution (excess kurtosis = 0.21; original data kurtosis = 0.68). For the German dataset, the 10^th^, 25^th^, 50^th^, 75^th^, and 90^th^ percentiles were 0.90, 1.40, 1.87, 2.37, and 2.65, respectively, compared with 0.70, 1.20, 1.80, 2.24, and 2.65 in the original dataset, indicating only slightly higher scores across most of the distribution in the German sample while the upper tail (90^th^ percentile) was identical.

**TABLE 2.** Item score distribution indicators for the German and the original sample (in this order: mean, standard deviation, skewness, excess kurtosis, item-drop Cronbach’s Alpha with 95% CI, and corrected item-to-total correlation).).

| Item | *M* | | *SD* | | *γ_1_* | | *Kurt* | | *α_drop_*  *[95%CI]* | | *R_corrected_*  *[95%CI]* | |
| --- | --- | --- | --- | --- | --- | --- | --- | --- | --- | --- | --- | --- |
|  | Ger | Orig | Ger | Orig | Ger | Orig | Ger | Orig | Ger | Orig | Ger | Orig |
| 1. Hand lowering | 3.6 | 3.6 | 1.3 | 1.2 | -0.91 | -0.88 | 0.30 | 0.51 | .68  [.62; .73] | .65 [.56;.72] | .53  [.44;.62] | .39  [.26;.49] |
| 2. Moving hands together | 3.1 | 3.1 | 1.5 | 1.3 | -0.53 | -0.62 | -0.59 | -0.21 | .70  [.64; .75] | .66 [58;.73] | .42  [.31;.51] | .29  [.15;.42] |
| 3. Mosquito hallucination | 1.5 | 1.7 | 1. | 1.5 | 0.62 | 0.48 | -0.67 | -0.79 | .70  [.64; .77] | .65 [.56;.72] | .41  [.32;.50] | .36  [.24;.46] |
| 4. Taste hallucination | 1.9 | 1.8 | 1.5 | 1.4 | 0.31 | 0.43 | -1.09 | -0.81 | .71  [.65; .75] | .65 [.57;.72] | .37  [.28;.46] | .35  [.23;.45] |
| 5. Arm rigidity | 2.9 | 3.2 | 1.6 | 1.4 | -0.38 | -0.67 | -0.97 | -0.47 | .66  [.59; .72] | .62 [.52;.70] | .60  [.53;.67] | .50  [.41;.59] |
| 6. Arm immobilization | 2.5 | 2.7 | 1.1 | 1.4 | -0.08 | -0.33 | -1.05 | -0.77 | .67  [.61; .73] | .62 [.51;.69] | .55  [.47;.63] | .54  [.44;.62] |
| 7. Music hallucination | 0.6 | 0.3 | 1.2 | 0.9 | 2.02 | 3.86 | 2.96 | 15.39 | .72  [.67; .77] | .67 [.58;.73] | .23  [.11;.35] | .30  [.16;.42] |
| 8. Negative visual hallucination | 0.3 | 0.4 | 1.0 | 1.2 | 3.30 | 3.23 | 10.50 | 8.91 | .73  [.67; .77] | .69 [.61;.75] | .19  [.08;.29] | .13  [-.03;.28] |
| 9. Amnesia | 1.2 | 1.7 | 1.3 | 1.4 | 0.79 | 0.41 | -0.42 | -0.93 | .71  [.65; .75] | .67 [.58;.73] | .35  [.24;.45] | .29  [.16;.40] |
| 10. Post-hypnotic suggestion | 0.8 | 1.0 | 1.3 | 1.5 | 1.53 | 1.06 | 1.46 | -0.25 | .74  [.69; .78] | .67 [.59;.73] | .14  [.04;.25] | .28  [.16;.40] |

Note. CIs were calculated using a bootstrapping procedure. Corrected item-to-total correlation means correlating an item with the scale score excluding that specific item's contribution.

**B) Exploratory Fator Analysis**

To examine the similarity of the latent structure between the German and original versions of the scale, exploratory factor analyses (EFAs) were conducted separately for each sample. Prior to factor extraction, Pearson correlation matrices were computed for the item responses. Scree plots based on the eigenvalues of the correlation matrices were inspected to evaluate the dimensionality of each dataset (Fig. 3). The scree plot for the original sample suggested a unidimensional factor structure. In contrast, the scree plot for the German sample did not exhibit a clear “elbow” point. For a conservative comparison of the factor structures, we extracted two factors in both samples, as the first two factors in the original sample had eigenvalues greater than 1 (Kaiser–Guttman criterion). EFAs were estimated with two factors in both samples using the minimum residual (*minres*) extraction method and an oblique (*oblimin*) rotation. Factor loadings were examined for both solutions (Table 3). Structural similarity between the German and original versions was quantified using Tucker's coefficients of factor congruence. The congruence coefficients for the first and second factors were .95 and .88, respectively, indicating very high similarity of the factor structures across the two samples.

**Figure 3**

*Scree plot for German and original sample.*

*Note*. The scree plot displays the eigenvalues associated with each extracted factor in descending order.

**TABLE 3.** Factor loadings and communalities in a two-factor structure for the German and the original sample.

|  | German sample | | | Original Sample | | |
| --- | --- | --- | --- | --- | --- | --- |
| Item | *F1* | *F2* | *h^2^* | *F1* | *F2* | *h^2^* |
| 1. Hand lowering | .79 | −.06 | .59 | .77 | −.10 | .52 |
| 2. Moving hands together | .70 | −.13 | .44 | .65 | −.08 | .37 |
| 3. Mosquito hallucination | .18 | .44 | .28 | −.02 | .71 | .48 |
| 4. Taste hallucination | .19 | .36 | .21 | .10 | .56 | .38 |
| 5. Arm rigidity | .72 | .14 | .61 | .59 | .23 | .55 |
| 6. Arm immobilization | .63 | .14 | .48 | .54 | .28 | .53 |
| 7. Music hallucination | −.08 | .49 | .21 | .01 | .34 | .12 |
| 8. Negative visual hallucination | −.07 | .37 | .12 | −.01 | .23 | .05 |
| 9. Amnesia | .08 | .42 | .21 | .33 | .16 | .19 |
| 10. Post-hypnotic suggestion | −.08 | .32 | .09 | .18 | .22 | .12 |

**C) PCS-GER wording of the questions**

1. **Hand lowering:**

Auf einer Skala von 0 bis 5, wie stark fühlten Sie, dass Ihre Hand schwerer wurde?

0 bedeutet, Ihr Arm war nicht schwerer als normalerweise und 5 heißt, Ihr Arm fühlte sich an, als ob ein schweres Objekt ihn herunterzieht

2. **Moving hands together:**

Sie wurden angewiesen, Ihre Hände vor Sich auszustrecken und Sich vorzustellen, eine imaginäre Kraft würde Ihre Hände zusammenziehen.

Auf einer Skala von 0 bis 5, wie stark war die Kraft zwischen Ihren Händen? 0 heißt, Sie fühlten gar keine Kraft und 5 heißt, Sie fühlten eine so starke Kraft, es kam Ihnen so vor, als ob Ihre Hände Magneten waren.

3. **Mosquito hallucination:**

Sie wurden darauf angewiesen zu bemerken, dass eine Mücke um Sie herum summt und wurden angewiesen, sie zu vertreiben.

Auf einer Skala von 0 bis 5, wie stark fühlten Sie die Präsenz der Mücke? Dabei heißt 0, Sie hatten gar nicht das Gefühl, dass eine Mücke da wäre und 5 heißt, es kam Ihnen so vor, als ob eine Mücke präsent wäre.

4. **Taste hallucination:**

Ihnen wurde gesagt, Sie würden einen süßen Geschmack in Ihrem Mund schmecken, dann einen sauren.

Auf einer Skala von 0 bis 5, wie stark war der süße Geschmack in ihrem Mund? 0 heißt, Sie schmeckten nichts und 5 heißt, Sie schmeckten einen starken Geschmack.

Auf einer Skala von 0 bis 5, wie stark war der saure Geschmack in Ihrem Mund? 0 heißt, Sie schmeckten nichts und 5 heißt, Sie schmeckten einen starken Geschmack.

5. **Arm rigidity:**

Ihnen wurde als nächstes gesagt, Sie sollen Ihren rechten Arm ausstrecken und bemerken, dass er steif wird und dann sollten Sie versuchen, den Arm zu beugen.

Auf einer Skala von 0 bis 5, wie steif fühlte sich Ihr Arm an? 0 heißt, Ihr Arm war nicht mehr steif als normalerweise und 5 heißt, sie konnten Ihren Arm gar nicht mehr beugen.

6. **Arm Immobilisation:**

Ihnen wurde gesagt, dass sich Ihre linke Hand sehr schwer anfühlt und Sie wurden angewiesen zu versuchen, Ihre Hand zu heben.

Auf einer Skala von 0 bis 5, wie stark war das Gefühl der Schwere in Ihrer Hand? 0 heißt, Sie spürten gar keine Schwere und 5 heißt, es fühlte sich so an, als ob ein schweres Objekt auf Ihre Hand drückt

7. **Music hallucination:**

Als nächstes wurden Sie gebeten, Ihre rechte Hand zu heben, wenn Sie eine Aufnahme von Happy Birthday hören können.

Auf einer Skala von 0 bis 5, wie klar hörten Sie die Musik? 0 heißt, Sie konnten gar keine Musik hören und 5 heißt, es klang so, als ob die Musik aus Lautsprechern kommen würde.

8. **Negative visual halluzination:**

Sie wurden angewiesen, Ihre Augen zu öffnen und ein Bild von zwei farbigen Bällen anzusehen. Sie haben daraufhin die Farbe der Bälle angegeben.

Auf einer Skala von 0 bis 5, wie unsichtbar war der dritte Ball? 0 heißt, Sie haben ganz klar drei Bälle gesehen und 5 heißt, Sie haben zwei Bälle gesehen. Die Zahlen in der Mitte heißen, Sie hatten Probleme, den dritten Ball zu sehen.

9. **Amnesia:**

Ihnen wurde gesagt, Sie können Sich an nichts mehr erinnern, was Sie während der Sitzung gemacht haben, bis Sie die Anweisung bekamen, Sich wieder erinnern zu können.

Auf einer Skala von 0 bis 5, wie schwer war es, Sich an Ereignisse zu erinnern, bevor Sie die Anweisung bekamen, dass Sie Sich wieder erinnern können? 0 heißt, Sie konnten Sich so erinnern wie sonst auch und 5 heißt, Sie hatten keine Erinnerungen mehr.

10. **Post-session suggestion:**

Ihnen wurde gesagt, Sie sollen die Leertaste 6-mal drücken, aber Ihnen wurde auch gesagt, Sie werden vergessen, dass das Ihnen aufgetragen wurde.

Auf einer Skala von 0 bis 5, wie stark war das Bedürfnis die Leertaste zu drücken? 0 heißt, Sie hatten gar kein Bedürfnis und 5 heißt, Sie hatten ein starkes Bedürfnis.

Auf einer Skala von 0 bis 5, wie sehr konnten Sie Sich an die Instruktionen erinnern, die Leertaste zu drücken? 0 heißt, Sie konnten Sich normal daran erinnern und 5 heißt, Sie hatten gar keine Erinnerungen mehr an die Instruktion.

**D) PCS-GER German text for the script read by the narrator.**

This is the translated text script read to the participant. Sections that are underlined are not read out loud but provide instructions to the narrator or structure. We have also included the start and end points of the used audio files that are available on the GitHub repository.

**Script**

**Start of audio file 1**

Sie werden nun einige Übungen erhalten, in denen Sie Ihre Vorstellungskraft einsetzen können, um das Erleben bestimmter Situationen zu simulieren. Ziel ist es herauszufinden, wie sehr Sie die Art und Weise kontrollieren können, in der Sie einige (einfache) Situationen erleben, wie beispielsweise eine Handbewegung.

So würde ich Sie nun zum Beispiel bitten Ihre Hand zu senken, und sich vorzustellen, dass Sie wie von selbst nach unten gezogen wird. Lassen Sie sich auf diese Vorstellung ein, bis es sich tatsächlich so anfühlt, als würde sie ohne ihr Dazutun nach unten gezogen werden. Konzentrieren Sie sich dabei auf Ihre Empfindungen und auf die vorgestellte Situation, damit Sie in diese Realität eintauchen können, so als würden Sie sich beispielsweise von einer Erzählung eines Films mitreißen lassen, als wäre diese real. Danach werde ich Sie bitten, zu bewerten wie stark Sie jede dieser Erfahrungen empfinden konnten, wie z.B. wie stark sich Ihr Arm von selbst zu bewegen schien.

Lassen Sie mich wissen, wenn Sie noch Fragen haben. Ihre Ergebnisse bei diesen Übungen hängen vor allem von Ihrer Bereitschaft ab sich darauf einzulassen und zu versuchen sich die Dinge, die ich Sie bitten werde, sich vorzustellen, so gut wie möglich zu visualisieren. Machen Sie bitte gut bei diesem Experiment mit, indem Sie versuchen,sich das, was ich Ihnen beschreibe, lebhaft vorzustellen.

Nun setzten Sie sich bitte bequem hin und legen Sie ihre Hände in den Schoß.

So ist es richtig.

Wir werden zum Aufwärmen eine einfache Imaginationsübung machen. Ich werde gleich anfangen von 1 bis 20 zu zählen. Während ich zähle, stellen Sie sich vor, dass Sie einige Treppenstufen heruntergehen... fühlen Sie, wie Sie auftreten und sehen Sie die Stufen... erleben Sie das Tempo auf jede erdenkliche Art und Weise... 1 ... Sie sind eine Stufe heruntergegangen. 2 ...Schritte. 3 ... 4... die die Stufen herunter gehen ... 5 ... ... 6... 7... Sie stellen fest, dass Sie sich in Ihre Phantasie vertiefen können, nichts wird Sie stören... Konzentrieren Sie einfach Ihre Gedanken auf meine Stimme und auf die Dinge, über die ich Ihnen sage, dass Sie an sie denken sollen. Es fällt Ihnen leicht, den Dingen, von denen ich Ihnen erzähle,zuzuhören und ihnen zufolgen. 8...9,10... Sie haben die Hälfte geschafft... 11... 12...13...14...15... obwohl Sie in ihrer Vorstellungskraft vertieft sind, können Sie meine Stimme deutlich hören. 16... 17...18...Sie sind voll auf die Schritte konzentriert. Sie werden viele Dinge erleben, von denen ich Ihnen sagen werde, dass Sie sie erleben sollen. 19 20. So ist es richtig. Nun werden wir mit unseren Übungen beginnen.

Am Ende dieser Standardprozedur werde ich Sie bitten, über Ihre Erfahrungen am Computer zu berichten.

Ich denke, jetzt können wir beginnen.

1. *Absenken der Hand*

Halten Sie nun Ihre rechte Hand auf Schulterhöhe ausgestreckt nach vorn, mit der Handfläche nach oben zeigend. Die rechte Hand gerade ausgestreckt vor sich, die Handfläche nach oben. So ist es richtig... Achten Sie sorgfältig darauf, wie sich die Hand nun anfühlt, was in ihr vorgeht. Achten Sie darauf, ob sie ein wenig taub ist oder kribbelt; achten sie auf die leichte Anstrengung, die notwendig ist um das Handgelenk nicht zu beugen; jeder Windhauch, den Sie auf ihr spüren. Achten Sie jetzt genau auf Ihre Hand. Stellen Sie sich vor, dass Sie etwas Schweresin Ihrer Hand halten... vielleicht eine schwere Bowlingkugel - etwas Schweres. Formen Sie Ihre Finger so, als hielten Sie diesen schweren Gegenstand, von dem Sie sich vorstellen, dass er sich in Ihrer Hand befindet. Sehr gut......Jetzt fühlen sich Hand und Arm schwer an, als ob das Gewicht sie nach unten drückt ...und während es sich immer schwerer anfühlt, beginnen Hand und Arm sich nach unten zu bewegen... als ob sie nach unten gezwungen werden... sie bewegen sich weiter nach unten... weiter nach unten... und weiter....mehr und mehr nach unten... schwerer... schwerer... der Arm wird immer müder und angestrengter....nach unten... langsam, aber sicher... nach unten, nach unten ..mehr und mehr nach unten, das Gewicht ist so groß, die Hand ist so schwer... Man spürt das Gewicht immer mehr. ..der Arm istzu schwer, um ihn zurückzuhalten... er geht immer weiter nach unten, nach unten... mehr und mehr nach unten...

*(Lassen Sie zehn Sekunden Zeit.)*

So ist es gut jetzt lassen Sie Ihre Hand wieder in ihre ursprüngliche Ruheposition zurückgehen. Sie haben wahrscheinlich viel mehr Schwere und Müdigkeit in Ihrem Arm erlebt, als wenn Sie sich nicht darauf konzentriert und sich nicht vorgestellt hätten, dass etwas versucht, ihn nach unten zu zwingen. Ihre Hand und Ihr Arm sind jetzt so, wie sie waren, sie fühlen sich weder müde noch angespannt an....

2. *Gemeinsam die Hände bewegen.*

Strecken Sie nun Ihre Arme vor sich aus, die Handflächen einander zugewandt, die Hände etwa schulterbreit auseinander. Halten Sie Ihre Hände etwa auf Höhe der Schultern, die Handflächen einander zugewandt. Ich möchte nun, dass Sie an eine unsichtbare Kraft denken, die auf Ihre Hände wirkt und sie zusammenzieht, so als ob eine Hand die andere anziehen würde. Denken Sie an Ihre Hände, wie diese zusammengezogen werden. Sie beginnen, sich aufeinander zu zubewegen... aufeinander zu... näher... immer näher... kommen immer näher... immer weiter und weiter aufeinander zu... mehr und mehr...

3. *Moskitohalluzination*

Sie haben mir sehr aufmerksam zugehört und gut aufgepasst. Es könnte sein, dass Sie die Mücke nicht bemerkt haben, die hier geschwirrt und gesungen hat, so wie es Mücken tun ... Hören Sie ihr jetzt zu... hören Sie ihr hohes Summen, wie sie um Ihre rechte Hand herumfliegt ... Sie landet auf Ihrer Hand ... vielleicht kitzelt es ein wenig... Sie fliegt wieder weg... Sie hören ihr hohes Summen ... Nun sitzt sie wieder auf ihrer Hand, es kitzelt wieder... vielleicht sticht sie... Sie mögen die Mücke nicht ... Sie möchten sie loswerden... Nur zu, streifen Sie sie ab ... werden Sie sie los, wenn sie Sie stört ...

(Warten Sie zehn Sekunden.)

Sie ist verschwunden ... Sie werden nicht mehr belästigt ... die Mücke ist verschwunden

4. *Geschmacks-Halluzination*

Als nächstes möchte ich, dass Sie an etwas Süßes in Ihrem Mund denken. Stellen Sie sich vor, Sie hätten etwas Süßes im Mund, zum Beispiel Zucker ... und wenn Sie an diesen süßen Geschmack denken, können Sie tatsächlich beginnen, den süßen Geschmack zu erleben und wirklich zu schmecken... Er mag anfangs nur schwach sein, aber er wird stärker... und stärker... Nun fangen Sie an, den süßen Geschmack in Ihrem Mund wahrzunehmen... Der süße Geschmack nimmt zu ... wird süßer ...und süßer ... Er wird stärker. Oft dauert es einige Augenblicke, bis ein solcher Geschmack seine volle Stärke erreicht ... Jetzt wird er deutlich stärker... stärker...

(Warten Sie zehn Sekunden.)

In Ordnung. Sie bemerken jetzt, dass mit diesem Geschmack etwas passiert. Er verändert sich. Sie beginnen nun, einen sauren Geschmack in Ihrem Mund wahrzunehmen... einen sauren Geschmack, als ob Sie etwas Zitrone im Mund hätten, oder etwas Essig... der Geschmack in Ihrem Mund wird saurer... und saurer... immer saurer...

(Warten Sie zehn Sekunden.)

In Ordnung. Jetzt verschwindet der saure Geschmack, und Ihr Mund fühlt sich genauso an wie vor der Erwähnung irgendeines Geschmacks. Ihr Mund fühlt sich jetzt normal an. Es ist jetzt alles wieder normal.

5. *Arm-Starre*

Bitte strecken Sie Ihren rechten Arm nach vorne aus und strecken Sie auch die Finge... So ist es richtig... Rechter Arm, nach vorne gestreckt. Stellen Sie sich vor, dass Ihr Arm immer starrer wird ... erstarrt ... sehr starr ... wenn Sie sich vorstellen, dass er erstarrt, werden Sie fühlen, dass er starrer wird ... starrer und starrer, als ob Ihr Arm in einer Schiene wäre, so dass sich der Ellenbogen nicht beugen kann ...starr ... starr und steif gehalten, so dass ersich nicht beugen kann. Ein fest geschienter Arm kann sich nicht beugen ... Ihr Arm fühlt sich steif an, als wäre er fest geschient ... Testen Sie mal, wie steif und starr er ist ... Versuchen Sie, ihn zu beugen ... versuchen Sie es...

(Warten Sie zehn Sekunden.)

Das ist gut so. Sie haben heut Gelegenheit, viele verschiedene Dinge zu erleben. Wahrscheinlich haben Sie bemerkt, wie Ihr Arm erstarrte, als Sie ihn für steifhielten, und wie viel Mühe es kostete, ihn zu beugen. Nun ist ihr Arm überhaupt nicht mehr steif. Bringen Sie ihn wieder in die richtige Position

6. *Immobilisierung des Arms (linke Hand)*

Jetzt sollte Ihre linke Hand in Ihrem Schoß liegen. Ich möchte, dass Sie jetzt über Ihren linken Arm und Ihre linke Hand nachdenken. Achten Sie genau auf sie. Sie fühlen sich taub und schwer, sehr schwer an. Wie schwer sich Ihre linke Hand anfühlt... Selbst wenn Sie darüber nachdenken, wie schwer Ihre linke Hand ist, wird sie immer schwerer und schwerer ... Ihre Hand wird immer schwerer ... schwerer und schwerer ... Ihre Hand wird immer schwerer, sehr schwer, als ob sie gegen Ihren Schoß gedrückt würde. Vielleicht würden Sie gern herausfinden, wie schwer Ihre Hand ist ... sie scheint viel zu schwer zu sein, um sie zu bewegen... aber obwohl sie so schwer ist, können Sie sie vielleicht ein klein wenig bewegen; aber vielleicht ist sie selbst dafür zu schwer ... Warum versuchen Sie nicht einmal herauszufinden, wie schwer sie ist ... Versuchen Sie, Ihre Hand hochzuheben, versuchen Sie es gern.

(Warten Sie zehn Sekunden.)

So ist es gut. Nun legen Sie Ihre Hand wieder in ihre Ruheposition zurück. Ihre Hand und Ihr Arm fühlen sich jetzt wieder normal an. Sie sind nun nicht mehr schwer.

7. *Musikhalluzination*

In wenigen Augenblicken wird eine Aufnahme von 'Happy Birthday to You' für Sie abgespielt. Wenn die Musik beginnt, wird die Lautstärke ganz heruntergedreht, und Sie werden es wahrscheinlich nicht oder nur sehr schwach hören können. Dann wird die Lautstärke erhöht, und ich möchte, dass Sie mir mitteilen, wann Sie eszufriedenstellend hören können, indem Sie Ihre rechte Hand hochhalten. Wenn Sie die Musik gut hören können, halten Sie Ihre rechte Hand hoch. In Ordnung? Und los geht's ... Die Aufnahme von 'Happy Birthday to You' wurde eingeschaltet. Das ist Stufe Eins.

(Warten Sie fünf Sekunden.)

Jetzt wird sie ein wenig lauter gestellt. Hier ist Stufe Zwei. Halten Sie Ihre Hand hoch, wenn Sie sie jetzt hören können.

(Warten Sie fünf Sekunden.)

Und jetzt lauter. Hier ist Stufe drei. (Warten Sie fünf Sekunden.) Und jetzt die lauteste Einstellung. Das ist Stufe Vier. Halten Sie Ihre Hand hoch, wenn Sie die Musik jetzt hören können.

(Warten Sie fünf Sekunden.)

Jetzt ist die Musik ausgeschaltet. Jetzt gibt es keine Musik mehr. Sie können Ihre Hand wieder in die Ruheposition zurückführen. Jetzt ... lehnen Sie sich einfach zurück.

8. *Negative visuelle Halluzination*

Während Sie bequem in Ihrem Stuhl mit geschlossenen Augen sitzen, wird ein Bild von zwei Bällen auf dem Bildschirm vor Ihnen angezeigt. Die beiden farbigen Bälle werden vor Ihnen auf dem Bildschirm angezeigt, so dass Sie sie deutlich sehen können. Gleich werde ich Sie bitten, die Augen zu öffnen. Sie werden nur zwei Bälle auf dem Bildschirm sehen... nur zwei Bälle...

(Zeigen Sie eine Folie mit drei farbigen Kugeln an.)

Okay, jetzt ist es an der Zeit, Ihre Augen zu öffnen, auf den Bildschirm zu schauen und die Eingabetaste zu drücken. Dort sehen Sie die beiden Bälle. Bitte merken Sie sich die Farbe der Kugeln, die Sie sehen. Merken Sie sich die Farbe der Kugeln, die Sie sehen, damit Sie sie später berichten können. Okay, schließen Sie jetzt Ihre Augen und...

(Entfernen Sie die Folie mit den drei farbigen Kugeln.)

Nun möchte ich Sie bitten, Ihre Augen zu öffnen und die Farbe der Bälle, die Sie gesehen haben, einzugeben

**End of audio file 1**

**Start of audio file 2**

*9. & 10.	Amnesie und Post-Sitzungs Suggestion*

Für die nächste Übung sollten Sie das Gefühl haben, dass es Ihnen schwerfällt, sich an all die Dinge zu erinnern, die ich Ihnen gesagt habe, und an all die Dinge, die Sie getan oder gefühlt haben, seit Sie Ihre Augen geschlossen haben. Tatsächlich werden Sie sich so sehr anstrengen müssen, sich an all diese Dinge zu erinnern, dass Sie es gar nicht versuchen wollen. Es wird viel einfacher sein, einfach alles zu vergessen, bisich Ihnen sage, dass Sie sich wieder erinnern können. Sie werden sich an nichts von dem erinnern, was Sie getan, gesehen oder gefühlt haben, seit Sie Ihre Augen geschlossen haben, bis ich Ihnen sage "Jetzt können Sie sich an alles erinnern!". Sie werden sich an nichts erinnern, was Sie bis dahin getan haben

Gleich werde ich Sie bitten, Ihre Augen zu öffnen. Dann erscheint auf dem Bildschirm die Meldung "Bitte warten". Wenn auf dem Bildschirm die Meldung "Bitte warten" erscheint, drücken Sie sechsmal die Leertaste"? Sie werden die Leertaste sechsmal drücken, aber Sie vergessen, dass ich Ihnen das gesagt habe, genauso wie die anderen Dinge. Bis ich Ihnen sage: "Jetzt können Sie sich an alles erinnern".

Öffnen Sie jetzt bitte Ihre Augen.

**End of audio file 2**

**Start of audio file 3**

Bitte geben Sie jetzt kurz eine Liste der Dinge ein, die seit unserem Beginn geschehen sind. Gehen Sie nicht ins Detail. Verbringen Sie zwei Minuten, nicht länger, mit dem Schreiben Ihrer Antwort. Ich gebe Ihnen Bescheid, wenn die Zeit abgelaufen ist.

**End of audio file 3**

(Warten Sie zwei Minuten.)

**Start of audio file 4**

Hören Sie meinen Worten aufmerksam zu. Jetzt können Sie sich an alles erinnern. Schreiben Sie eine Liste mit allem, woran Sie sich jetzt erinnern, woran Sie sich vorher nicht erinnert haben. Bitte gehen Sie nicht ins Detail. Verbringen Sie zwei Minuten, nicht länger, mit diesem Abschnitt. Auch hier werde ich Sie wissen lassen, wenn die Zeit abgelaufen ist.

**End of audio file 4**

(Warten Sie zwei Minuten.)

**Start of audio file 5**

Drücken Sie jetzt bitte Return. Auf den folgenden Bildschirmen finden Sie eine Liste der spezifischen Ereignisse, die Ihnen während der Sitzung vorgeschlagen wurden. Bitte lesen Sie die Anweisungen und beantworten Sie dann die Fragen. Arbeiten Sie sie bis zum Ende durch.

**End of audio file 5**
