# Supplementary material for: Norms for a German version of the Phenomenological Control Scale: measuring the capacity for creating and controlling experience
Source: Neurosci Conscious. 2026 Oct 31;2026(1):niag073. doi: 10.1093/nc/niag073 (PMC13723268; doi:10.1093/nc/niag073)
Supplement: Supplementary file 1 [file supplementary_materials_niag073.zip › OPEN SCIENCE BADGE APPLICATION FORM.pdf]

OPEN SCIENCE BADGE APPLICATION FORM

Open Data Badge

Please provide the URL, DOI, or other permanent path for accessing the data in a public, open access repository.

https://doi.org/10.5281/ZENODO.20068742

Is there sufficient information for an independent researcher to reproduce the reported results? If no, explain.

Yes

Open Materials Badge

Please provide the URL, DOI, or other permanent path for accessing the materials in a public, open access repository.

https://doi.org/10.5281/ZENODO.20068742

Is there sufficient information for an independent researcher to reproduce the reported methodology? If no, explain.

Yes

Preregistered Badge

Please provide the URL, DOI, or other permanent path to the registration (and, if applicable, the analysis plan) in a public, open access repository.

https://osf.io/b7yfx

Was the plan preregistered prior to the examination of the data or observing the outcomes? If no, explain.

Yes

Were there additional registrations for the study other than the one reported? If yes, provide links and explain.

No

For Preregistered and Analysis plan badge: were there any changes to the preregistered analysis plan for the primary confirmatory analysis? If yes, explain.

Just additional analyses

For Preregistered and Analysis plan badge: are all of the analyses described in the registered plan reported in the article? If no, explain.

Yes
